# Unusual Presentation of Infective Endocarditis Following a Prostatic Urethral Lift

**DOI:** 10.7759/cureus.26919

**Published:** 2022-07-16

**Authors:** Suchit Chidurala, Manikumar Bheemarasetti

**Affiliations:** 1 Natural Sciences, University of Texas at Austin, Austin, USA; 2 Interventional Cardiology, Texas Health Heart and Vascular Hospital Arlington, Arlington, USA

**Keywords:** adult cardiac surgery, infectious disease, prostatic urethral lift, infective endocarditis, atypical presentation

## Abstract

Infective endocarditis is a serious inflammation of the inner lining of the heart. It is caused by pathogens entering the bloodstream and infecting the endocardium. We demonstrate a unique presentation of infective endocarditis following a prostatic urethral lift. The low index of suspicion and atypical symptoms prevented early diagnosis of the disease, leading to life-threatening complications and valve replacement surgery. Understanding unusual presentations of infective endocarditis can increase the index of suspicion in outpatient settings, leading to early diagnosis and preventing fatal complications.

## Introduction

Infective endocarditis (IE) is an infection of the inner lining of the heart by pathogens that enter the bloodstream [1]. Early diagnosis of IE is essential to improve clinical outcomes and survival; however, due to the subtle symptoms, diagnosis is often delayed [1]. IE typically presents with symptoms such as fever, murmur, and malaise; has risk factors of prior endocarditis, congenital heart disease, poor dental hygiene, and prosthetic valves; and is more likely with a medical history including IV drug use, rheumatic fever, or recent cardiac/dental surgical procedures [1-5]. 

A new minimally invasive technique that can treat benign prostate hyperplasia (BPH) is the UroLift device [6]. Formally known as a prostatic urethral lift (PUL), this procedure entails transurethral delivery of implants with ends anchored to the prostate capsule and urethra, retracting the lateral lobe of the prostate from the urethral lumen [7]. Hereby, we discuss a unique presentation occurring after a PUL that resulted in a diagnosis of subacute IE leading to aortic and mitral valve replacement. 

## Case presentation

**Figure 1:**
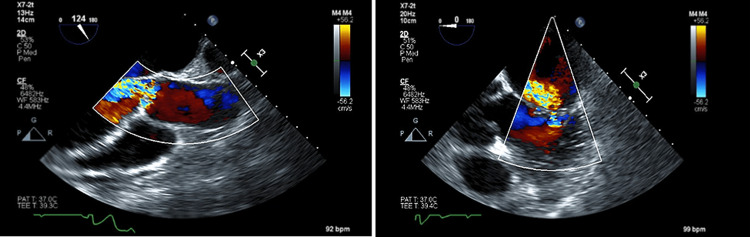
On the left, a TEE depicts moderate to severe aortic valve regurgitation. On the right, a TEE displays mild mitral valve regurgitation.

**Figure 2:**
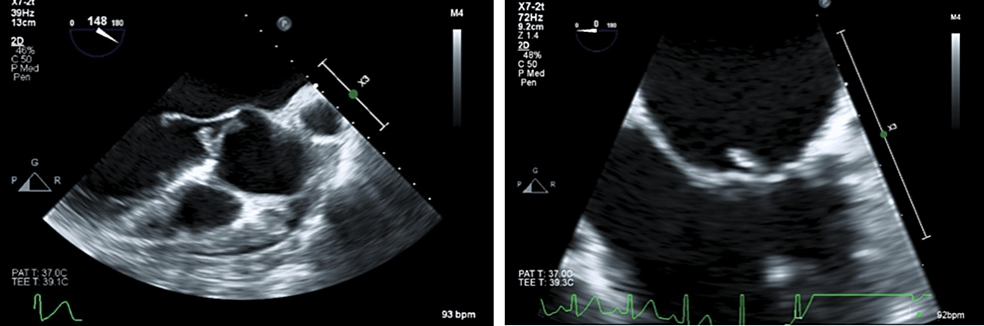
Aortic valve vegetation shown on the left and mitral valve vegetation shown on the right viewed on a TEE

A 59-year-old Caucasian male with a past medical history of anxiety, Barrett’s esophagus, chronic anemia, gastroesophageal reflux disease, hypertension, smoking, BPH, and urinary tract infection (UTI) status post PUL and intermittent self-catheterization presented to the cardiology clinic for exertional dyspnea, weight loss, and preoperative evaluation for an upper gastrointestinal endoscopy. At that time, he was taking diazepam, diltiazem, doxazosin, ferrous sulfate, and omeprazole for his conditions. On examination, he was found afebrile and tachycardic at 116 beats per minute. He also had bilateral leg swelling. A transthoracic echocardiogram (TTE) in the clinic showed preserved ventricular function with an ejection fraction of 55%-60%, grade three diastolic dysfunction, moderate aortic insufficiency, and moderate mitral regurgitation. He was then admitted to inpatient for further management of acute congestive heart failure and was started on intravenous (IV) diuretics. His initial lab work showed a normal white cell count but hemoglobin of 8.8 g/dL. A subsequent transesophageal echocardiogram (TEE) revealed moderate-to-severe aortic valve regurgitation and an eccentric aortic regurgitation jet directed at the mitral valve with mild mitral regurgitation as shown in Figure 1. In addition, 1.5 cm x 1.2 cm vegetation on the aortic valve and small mitral valve vegetation were found as displayed in Figure 2. At this point, the likelihood of IE was very high, so we consulted the Infectious Disease (ID) physician, who immediately ordered blood cultures. The cultures grew *Enterococcus faecalis* susceptible to ampicillin with a minimum inhibitory concentration (MIC) less than or equal to two and vancomycin with a MIC of one. The antibiotic susceptibility test (AST) also showed sensitivity to gentamicin synergy. The patient was diagnosed with subacute IE and immediately started on IV vancomycin.

After questioning, the patient said that he was referred to a Urologist six months ago for urinary retention, nocturia, and frequent urination 16-20 times a day. The urologist diagnosed him with BPH and recommended a PUL to resolve symptoms. The patient started to intermittently self-catheterize upon urologist advice and underwent a PUL a month after the urologist consultation. After the procedure, the patient continued to self-catheterize but had recurrent UTIs. He received three courses of oral cefuroxime 500 mg twice daily (BID) for 10 days with no improvement. Due to worsening UTI symptoms, he went to Urgent Care one month prior to admission and was given nitrofurantoin 100 mg BID for 10 days after his urine culture grew *E. faecalis* per patient. The patient did not know about any antibiotic susceptibilities taken at the urgent care. Despite the antibiotic courses, he still reported recurrent dysuria and urinary retention. While taking nitrofurantoin, he started developing severe shortness of breath (SOB) and activity intolerance that resolved with rest. Over the following weeks, he had worsening SOB in addition to a 15-pound weight loss due to a loss of appetite. After visiting his primary care physician for these complaints, his blood test resulted in hemoglobin of 9.4 g/dL. He went to the Emergency Department (ED) where he was diagnosed with anemia without acute bleeding; given ferrous sulfate; and referred to multiple specialists. This led to his cardiology clinic consultation and subsequent hospitalization as explained above.

**Figure 3:**
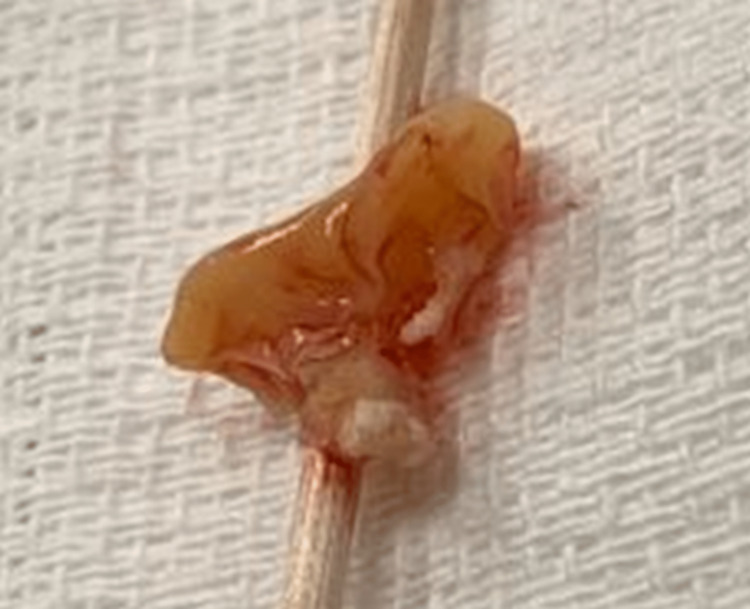
Aortic valve vegetation resected from the patient

After being diagnosed with subacute IE in the hospital, cardiothoracic (CT) surgery was consulted. He had persistently positive blood cultures despite antibiotic therapy. In addition, the patient had progressive dyspnea, worsening pulmonary infiltrates, and congestive heart failure. The CT surgeon recommended aortic and mitral valve replacements. For preoperative planning, he had a cardiac catheterization which showed nonobstructive disease. Due to his iron deficiency anemia, he also had an esophagogastroduodenoscopy and colonoscopy, which did not show any obvious bleeding source. Four days later, he underwent mitral and aortic valve replacements with the implantation of a 27 mm Masters mechanical heart valve and 21 mm St. Jude Regent mechanical heart valve, respectively. Intraoperatively, aortic vegetations on the left coronary and noncoronary cusps were found and shown in Figure 3. The mitral valve vegetations were mobile and seen on both the anterior and posterior mitral valve leaflets. There were also small nodules on the posterior subvalvular apparatus. Both valves and the subvalvular apparatus were resected and replaced with prosthetic valves. Histological sections revealed valve tissue with coagulative necrosis, mixed acute and chronic inflammation, and focal clusters of bacterial cocci. He was continued with ampicillin and gentamicin. After the surgery, the patient had a near-complete return to baseline health and was discharged with the recommendation to continue antibiotics until six weeks after the date of the surgery.

## Discussion

IE is a relatively rare but serious condition due to its high morbidity and mortality [5,8,9]. With a mortality of 20%-25% per year [4,10], IE is one of the most common life-threatening infections [11]. IE is widely diagnosed according to the modified Duke criteria [2,3], however, due to the heterogeneity of the disease and variable clinical presentations, there are shortcomings in the diagnostic criteria [12]. Thus, it is imperative to acknowledge unusual manifestations of the disease, as seen in this case, to circumvent these shortcomings and prevent a delay in the diagnosis of this life-threatening condition. 

*E. faecalis* is the third leading cause of IE causing 5%-15% of IE and 90% of enterococcal infections [2,4,13-17]. Enterococci are a group of microorganisms normally found in the gut microbiota, oral cavity, and vaginal vault [2,18,19]. However, they are opportunistic pathogens and a major cause of UTIs, intra-abdominal infections, bacteremia, and IE [13,18,19]. This coupled with their resistance to many antibiotics poses enterococci as a major clinical problem [2,4,13]. The most common entry portal for *E. faecalis* is the genitourinary system where diagnostic and therapeutic instrumentations increase the risk of infection [1,2,4,13,16,17,19]. In fact, enterococci are responsible for 15% of catheter-associated UTIs through a mechanism dependent on their endocarditis and biofilm-associated pilus [20]. Due to this prevalence, the likely source of the patient’s infection was cystoscopy, self-catheterization, or PUL. Furthermore, the patient’s persistent UTIs after the procedure despite antibiotic treatment can be explained by the antibiotic-resistant nature of *E. faecalis*. While we do not know the antibiotic susceptibilities from the urgent care visit, the AST ordered by the ID physician did not report susceptibility to nitrofurantoin. Proper identification by urine and blood culture with sensitivities should have been done to prevent bacteremia and to give appropriate treatment. 

The most serious complication caused by an *E. faecalis* infection is endocarditis [13] secondary to bacteremia. Bacteremia resulting from an *E. faecalis* infection occurs due to inadequate antibiotic treatment, allowing the bacteria to enter the bloodstream. Since *E. faecalis* has high levels of antibiotic resistance, bacteremia is a common complication. *E. faecalis* antibiotic resistance is believed to be linked to the enterococcal surface protein gene product, a surface protein involved in adhesion, colonization evasion of the immune response, and biofilm formation [18]. Because the culture was not susceptible to nitrofurantoin as per the AST, the antibiotics prescribed to the patient for his recurrent UTIs at the urgent care were not efficacious against the *E. faecalis* infection, resulting in bacteremia. Early cultures must be taken to determine appropriate antibiotics to prevent the spread of infection.

IE caused by *E. faecalis* has a high mortality rate of 11%-35%, which remains unchanged despite medical advancements [4,15]. However, presentations of *E. faecalis* IE have variable symptoms and paint a complex clinical picture, making it difficult to diagnose [3,14]. With a low diagnostic yield in the early phases, *E. faecalis* IE risks overlooking serious infection [2,14]. Echocardiography is essential to address the delayed diagnosis of *E. faecalis* IE [14]. While TTE is the first diagnostic tool, TEE might serve as a better tool due to its better sensitivity toward finding vegetations on a native valve (75% with TTE and 85%-90% with TEE) [2]. Treatment of *E. faecalis* IE is also difficult due to the antibiotic-resistant nature of the bacteria. Typically, two drugs are required for effective treatment, and surgery is often required to remove the infected valve [2,13]. A cell wall active agent like penicillins in combination with an aminoglycoside is the standard of care for *E. faecalis* IE, with gentamicin being preferred over streptomycin due to its greater synergistic effect with penicillins [11,13]. The surgery rates for *E. faecalis* IE range from 20% to 40% and are indicated by severe valve regurgitation, progressive heart failure, and large vegetations with a high risk of embolism [2].

We discuss a unique presentation of subacute IE by *E. faecalis* resulting in a delayed diagnosis due to subtle symptoms, unusual medical history, and low index of suspicion. Despite not having any risk factors for the disease or typical medical history associated with the disease, the patient developed IE. Fortunately, a TEE revealed vegetation, and the patient was promptly treated with appropriate antibiotics and valve replacement surgery. The route of infection was likely due to recent urinary procedures including a cystoscopy, self-catheterization, and/or the PUL. The prescribed antibiotics were not effective, leading to bacteremia and subacute IE by *E. faecalis*. Vancomycin, ampicillin, and gentamicin were administered at the hospital as per the results of the *in vitro* AST, which is a typical regime to treat IE. The combination of appropriate antibiotics and surgery led to a return to baseline health for the patient. The unique presentation of this case provides insight into the variable symptoms and history associated with subacute IE. It hopes to provide an understanding of the heterogeneity of this life-threatening infection to prevent a delayed diagnosis and severe complications. Familiarity with different presentations of subacute IE will help adjust diagnostic criteria to promote timely diagnosis and improve the relatively high and unchanging mortality of the infection.

## Conclusions

In conclusion, this case report presents the importance of early intervention to prevent life-threatening complications. With *E. faecalis* a not uncommon cause of UTIs, especially after urological procedures, urine and/or blood cultures with sensitivities should be done early on to prevent the spread of infection, especially if urinary symptoms persist after an antibiotic regime. Additionally, despite the unusual presentation, the symptoms presented by the patient should have raised suspicion of IE. Understanding unusual presentations will increase the suspicion of IE in outpatient settings, preventing delayed diagnosis.
